# Neurobehavioral, Neuromotor, and Neurocognitive Effects in Agricultural Workers and Their Children Exposed to Pyrethroid Pesticides: A Review

**DOI:** 10.3389/fnhum.2021.648171

**Published:** 2021-07-16

**Authors:** Boris Lucero, María Teresa Muñoz-Quezada

**Affiliations:** The Neuropsychology and Cognitive Neurosciences Research Center (CINPSI Neurocog), Faculty of Health Sciences, Universidad Católica del Maule, Talca, Chile

**Keywords:** neurotoxics, pesticides, pyrethroids, occupational health, neurocognitive effects, agricultural workers

## Abstract

In recent years, pyrethroids have emerged as a less toxic alternative to eliminate insect pests. However, some animal studies and studies with children show that these pesticides are toxic and lead to neurobehavioral effects similar to other pesticides, such as organophosphates. The purpose of this review was to systematize the epidemiological scientific evidence about the neurobehavioral, neuromotor, and neurocognitive effects in agricultural workers and their children exposed to pyrethroid pesticides. We conducted two searches (with different terms) in PubMed and Scopus databases, including articles in Spanish and English language on the effects of occupational exposure to pyrethroid pesticides associated with neurobehavioral, neuromotor, and neurocognitive functioning of agricultural workers and their children. There were no filters by year, and the search included studies till march 2021. To develop the search, we followed the recommendations contained in the PRISMA guidelines and the PICO strategy. The results show that in 66.6% of the studies reviewed (8 of 12 studies), agricultural workers or their children occupationally exposed to pyrethroid pesticides have a higher risk of presenting difficulties in their neurocognitive, neuromotor, or neurobehavioral performance, mainly associated with attention, processing speed (linked to hand-eye coordination), and motor coordination. There are still few studies that address this issue. However, the quality of most of the research conducted (83% intermediate or high quality) confirms the risk for neurobehavioral health in agricultural workers due to occupational exposure to pyrethroids. More research is required evaluating the exposure to pyrethroids, including biomarkers and validated neurobehavioral and neuromotor tests, in addition to evaluating the effect of simultaneous exposure to other hazardous pesticides. Assuming that the use of pyrethroids is increasing considerably and faster than the scientific evidence, it is suggested as a precautionary principle to regulate, more strictly, the sale of pyrethroids and other pesticides.

## Introduction

Neurotoxins are chemical substances external to the body, synthetic in general, that cause structural, biochemical, and functional damage to the central nervous system, peripheral nervous system, and sensory organs (Bai et al., 2020). Therefore, exposure to neurotoxins can cause sensory, motor, and neurocognitive disorders (Fishbein et al., 2019; Pérez-Fernández et al., 2019). Among neurotoxic substances, pesticides have been identified as one of the most frequent causes of neurocognitive disorders in agricultural workers (Muñoz-Quezada et al., 2016; Voorhees et al., 2017). Currently, occupational exposure to pesticides without adequate protective measures is identified as a public health issue (Muñoz-Quezada et al., 2017). The greater resistance to pests and the demands of the food industry have led to an increase in the sale and application of pesticides worldwide (Muñoz-Quezada et al., 2020). It means a higher exposure for workers, which added to their low educational attainment, lack of training, low-risk perception, low use of personal protective equipment, poor working conditions, inadequate supervision, biological susceptibility, and other associated environmental variables, have caused several occupational diseases related to acute or chronic pesticide poisoning (Bradman et al., 2009; Orozco et al., 2011).

Although high doses are required to cause acute poisoning or death from pesticide exposure, chronic neurodegenerative effects can occur associated with repeated exposure to low doses over time. The most common insecticides are organochlorines, organophosphates, carbamates, and pyrethroids. These pesticides generally affect the dynamics of neurotransmitters in synaptic communication or the conduction of action potential in the activity of ion channels of neurons (Hansen et al., 2017).

Since the last decade of the 20th century, there is sufficient evidence of the effects on neurodevelopment and neurocognitive performance caused by insecticides, especially organophosphates and organochlorines. Currently, organochlorine pesticides are banned worldwide due to their high toxicity and persistence in the environment. Although organophosphate and carbamate pesticides are less persistent, they have been increasingly restricted in their sale and use and are classified as high or moderately hazardous (Kori et al., 2018a). In addition, the demanding restrictions that developed countries have placed related to pesticide residues on imported agri-food products have slightly decreased their use. Several systematic reviews and meta-analyses have addressed the effects of organophosphate and carbamate pesticides on the nervous system in humans (Takahashi and Hashizume, 2014; Muñoz-Quezada et al., 2016).

On the other hand, synthetic pyrethroid pesticides were created in the mid-20th century based on natural chrysanthemum pyrethrins that were historically used by various countries in Asia, Africa, and Europe (Kolaczinski and Curtis, 2004). They appear as the alternative for organophosphates and carbamates, with supposedly less persistence and less toxicity. At present, they are used massively in agriculture and are the most sold for household use, either for the control of pests indoors or for the control of pediculosis in humans and mites in domestic animals. However, recent studies have shown adverse neurological consequences in mammals, and epidemiological studies have shown an association between environmental exposure to these pesticides and poorer neurocognitive performance (Babina et al., 2012).

The pyrethroid pesticides that are usually sold and recommended for application at home, because of their supposed relatively non-toxicity to humans, are made from three possible compounds: deltamethrin, permethrin, and cypermethrin (Chrustek et al., 2018). However, recent studies show that they are not entirely harmless to human health since they are considered neurotoxic substances, and each one has been identified with specific adverse effects: permethrin affects fertility, the immune system, enzymatic activity, cardiovascular, and hepatic metabolism; Deltamethrin induces inflammation, nephrotoxicity, and hepatotoxicity; and Alpha-cypermethrin can impair immunity and increase glucose and lipid blood levels.

According to the WHO (2009) recommended classification of pesticides by hazard, pyrethroids can be classified into various levels: slightly hazardous (Group III, blue label), moderately hazardous (Group II, yellow label), and highly hazardous (Ib, red label). The mechanism of action of pyrethroids in the nervous system is through the interaction with sodium channels and the induction of prolonged depolarization in neuronal cells. Based on acute intoxication symptoms, two types of pyrethroids can be distinguished: (a) Type I, which changes the sodium channels during their opening and closing in the neuronal membranes. It generates tremors throughout the body, aggressive behavior, hypersensitivity, and motor ataxia, and (b) Type II, which affects the sodium, chloride, and GABA channels, causing salivation and motor dysfunction (Chrustek et al., 2018).

The purpose of this review was to systematize the epidemiological scientific evidence of the PubMed, Scopus, and Scielo databases on the effects of occupational exposure to pyrethroid pesticides on neurobehavioral, neuromotor, and neurocognitive functioning of agricultural workers. This contribution could be useful for making the available evidence more accessible to decision-makers, the scientific community, and the field of public health and summarizing current research gaps and new evidence about neurocognitive outcomes related to occupational exposure to pesticides.

## Methods

**Figure 1:**
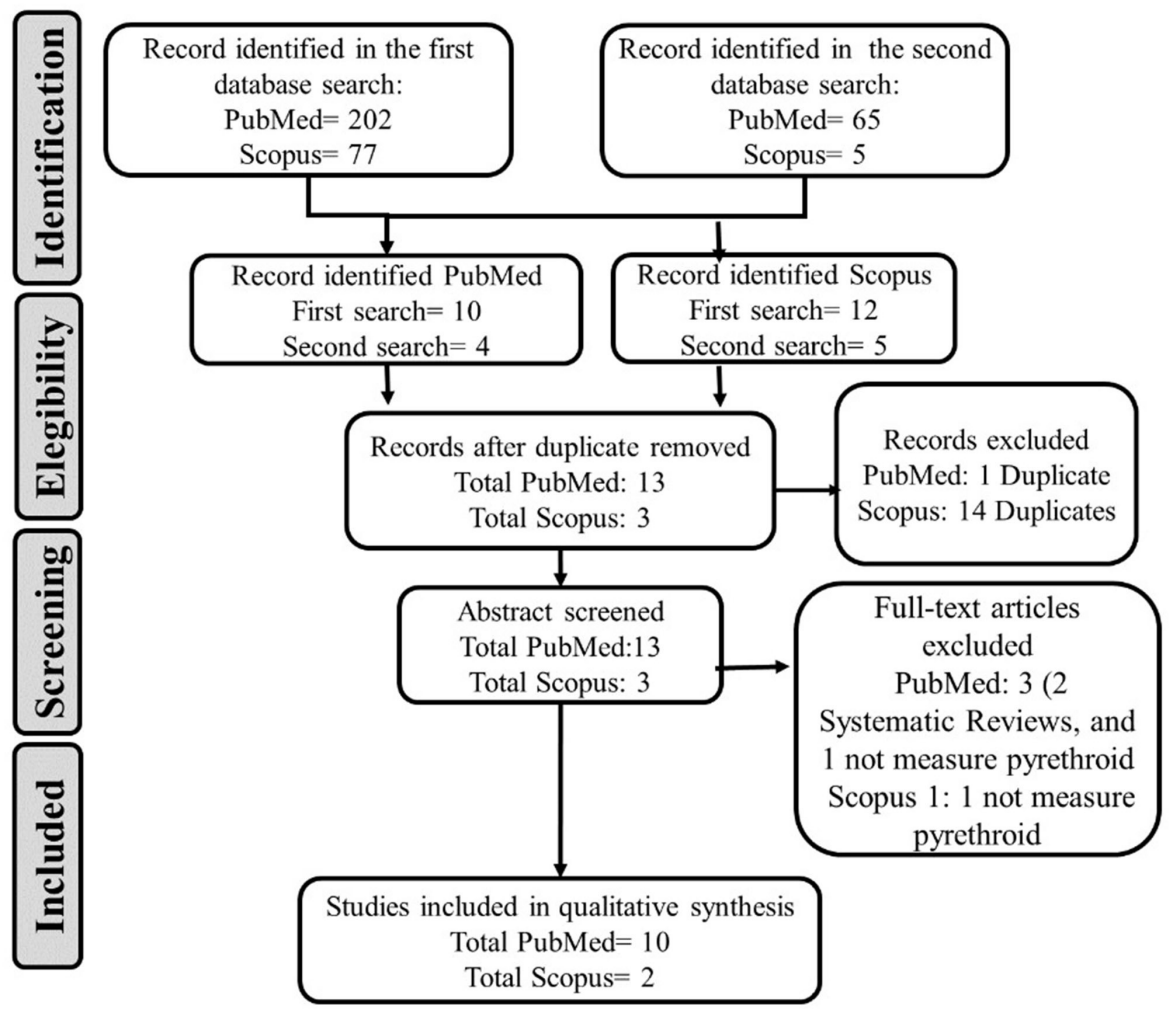
PRISMA flow diagram adapted from the systematic bibliographic review process carried out and its results.

The search for scientific publications in this review was made using the PubMed and Scopus databases (Figure 1) following the PRISMA guidelines (Moher et al., 2015).

Two searches were conducted in the PubMed Advanced Search Builder, Scopus, and Scielo databases, using the “All Fields” option to add terms. The search terms entered in the first search on the three databases were as follows: (pyrethroid) AND (health) AND (workers). In the second search, also including the three databases, the terms entered were as follows: (pyrethroid) AND (neurobehavioral). In the Scielo database, no studies related to pyrethroid exposure and health effects were found. Original publications in English, Spanish, or Portuguese were considered. Inclusion criteria were as follows: (a) assessment of chronic exposure to pyrethroids pesticides; (b) assessment of neurobehavioral, neuromotor, and neuropsychological functioning; and (c) adolescent or adult farmers and children of agricultural workers. There were no search filters by year, and the revision included articles published until March 2021.

Publications associated with systematic reviews, meta-analysis, case series reports, and regulatory or economic reports were excluded. The principal author of the review carried out the search and selection process. Both authors carried out the analysis of the selected articles independently. In PubMed, the first search identified 202 articles, with 10 articles meeting the inclusion criteria. The second search identified 65 articles, with four articles meeting the inclusion criteria and one duplicated from the previous search. Therefore, in PubMed, 13 studies were chosen for review. In Scopus, the first search identified 77 articles, with 12 articles meeting the inclusion criteria. In the second search, five articles met the inclusion criteria. The number of articles duplicated from Scopus was 14 (including the matching with PubMed database), so three studies were considered for review. After excluding the articles that did not include pyrethroids exposure measures, 10 studies in PubMed and 2 from Scopus were selected for synthesis (*n* = 12). The studies were organized according to the evidence of neurobehavioral, neuromotor, and neuropsychological effects associated with pyrethroid pesticide exposure (Table 1). Effects on the peripheral nervous system in motor performance were also considered because these neurological disorders affect neurocognitive functioning, mainly in fine motor coordination.

**Table 1:** Evidence of exposure to pyrethroids and neuro outcomes in adolescent and adult farmers and children of agricultural workers.

**Exposed participants (n), country**	**Intervention/** **Exposure to pyrethroids**	**Comparison/Control (*n*)**	**Effect and assessment instrument**	**Outcome (Neurobehavioral and neurocognitive effects) (Yes/No)**	**Design**	**Study qualification**	**References**
Male farm workers (*n* = 248), India	Exposure questionnaire: methods of pesticide use, health status, information about pesticide use such as crop, pesticide name, among other variables	There is no control group. The study compared: residence in farm vs. colony. Storage of pesticide in farm vs. home. Medium and high chronic–exposed farm workers.	Questionnaire that collected symptoms (self-reported): Stress, anxiety, depression, sleep problems, confusion, memory problems among others.	Yes	Descriptive	Intermediate low	Kori et al., 2018b
Male farm workers (*n* = 224), France.	Occupational history followed by interviews (exposure = pesticides used, insecticides, herbicides & fungicides, years used, pesticides containers, packages, and farming calendars)	Case = 224 Controls = 557	Questionnaires by a Mutualité Sociale Agricole Physician (Treated or hospitalized for depression).	No (Pyrethroid and others insecticide, yes for herbicides)	Case control	Intermediate	Weisskopf et al., 2013
Pesticide applicators (*n* = 30), Saudi Arabia.	Exposure questionnaire: Application of a mixture of type I (bifenthrin and bioallethrin) and type II (lambda-cyhalothrin, deltamethrin, cyphenothrin, and cypermethrin) pyrethroids	Non applicators from an administrative staff of the vector control unit in Jazan region, Arabia	Neurological symptoms (Q16 questionnaire); Neurobehavioral perfomance (BARS).	Yes	Cross-sectional	Intermediate	Ismail et al., 2018
Children of agricultural workers exposed to pesticides by living in a large-scale banana plantations (*n* = 38) or a small-holders who grew plantains (*n* = 48), Costa Rica.	Urine metabolites of pyrethroids (3-phenoxybenzoic acid, 3-PBA)	Children of agricultural workers living in a small-holders who grew mainly organically (*n* = 54)	Intellectual ability (WISC-IV); Behavioral problems (CPRS-R); Sensory function (LDD-15); Perception and memory (ROCF); Verbal memory and learning abilities (CAVLT-2); Visual-motor coordination (DTVP-2); Fine motor fuctioning (WRAVMA); Psychomotor speed (RTT).	Yes	Cross-sectional	Intermediate-high	van Wendel de Joode et al., 2016
18 children whose parents worked in their own small conventional coffee farms, Costa Rica	Urine metabolites of pyrethroids (3-phenoxybenzoic acid, 3-PBA)	17 children whose parents worked in organic coffee plantation	Neurobehavioral perfomance (BARS).	No	Cross-sectional	Intermediate	Lu et al., 2009
Pyrethroid pesticide applicators (*n* = 428), Iowa & North Carolina, USA	Exposure questionnaire: Mixture or applied 50 specific pesticides, including pyrethroids, reported in 1993–1997.	1,350 exposed to any type of pesticides (including pyrethroids)	Screening for dream enacting behavior (DEB) during REM sleep through a questionnaire, reported in 2013–2015.	Yes	Longitudinal	Intermediate	Shrestha et al., 2018
Agricultural workers applying various pesticides, including pyrethroids (*n* = 218), China	Written record for one year (2012) on the use of pesticides (chemical name, active ingredient percentage, amount of each pesticide product, date and duration of each spray)	There is no control group, however, pyrethroid applicators were compared with applicators of other pesticides	22 parameters of peripheral nerve conduction were examined using the surface electrodes with standard placement. Two rounds of conventional nerve conduction (year 2012).	No	Longitudinal	Intermediate-High	Zhang et al., 2018
Sprayers of the public vector control programs, only had used pyrethroids (*n* = 58), Bolivia	Structured interview	Sprayers had used various pesticides (*n* = 62)	Neuromotor and neurocognitive was assessed using the computerized Behavioral Assessment and Research System and CATSYS.	Yes	Cross-sectional	Intermediate-high	Hansen et al., 2017
224 farmers, short and medium term pesticide exposures, China	Records of all pesticides they applied (including pyrethroids).	There is no control group, however, pyrethroid applicators were compared with applicators of other pesticides	Conventional nerve conduction.	Yes	Longitudinal	High	Huang et al., 2016
121 women farmers, South Africa	Pyrethroid metabolite concentrations urinary (3PBA; 4F3PBA; DBCA, and the cis- and trans isomers of 2,2-dichlorovinyl-2,2-dimethylcyclopropane-1-carboxylic acid.	90 women from neighboring towns.	Neurological symptoms (Q16 questionnaire).	Yes	Cross-sectional	High	Motsoeneng and Dalvie, 2015.
Twenty-four children from a rice farming community (exposed), Thailand.	For pyrethroid metabolites, two metabolites, 3-phenoxybenzoic acid (3PBA) and cis/trans-2,2-(dichloro)-2-dimethylvinylcyclopropane carboxylic acid (DCCA).	29 from an aquaculture (shrimp) community (control)	Neurobehavioral perfomance (BARS).	No	Longitudinal	High	Fiedler et al., 2015
Full-time farmers (*n* = 398), Philippines	Exposure questionnaire to organophosphate, Pyrethroid, Carbamat, Other pesticides)	137 part-time farmers	The neurologic examination assessed all cranial nerves (I–XII). These cranial nerves were assessed by a neurologist by classifying clinical findings as abnormal or normal.	Yes	Cross-sectional	Intermediate low	Lu, 2009

The selected studies summarized in Table 1 were characterized according to PICO items (patient/participant, intervention, comparison, and outcomes), detailing the study design, sample size, exposure assessment, effect measurement, and control for confounding factors and biases (Pollock and Berge, 2017). The quality of each article was evaluated following the criteria already validated in previous reviews (Muñoz-Quezada et al., 2016), and each of the PICO items was assessed.

## Results

This review included 12 studies, of which, two were conducted in Africa, three in Latin America, five in Asia, one in Europe, and one in the USA (Table 1). Three studies were carried out with children of agricultural workers, one in women farmers, three with male farmworkers, and five with pesticide applicators. One article used a descriptive design, six studies used a cross-sectional epidemiological design, one was a case-control, and four studies used a longitudinal design. Four studies measured exposure to pyrethroids through urine biomarkers with specific pyrethroid metabolites, and five studies measured exposure through questionnaires, interviews, and records. The outcome was assessed in five studies with validated neurobehavioral or neurocognitive tests. Five studies evaluated effects through questionnaires about symptoms and two studies through peripheral nerve conduction with electrodes. A study evaluated neurobehavioral effects with a self-report questionnaire associated with symptoms of depression, stress, anxiety, and sleep problems, and another investigation evaluated the presence of neuromotor and balance disorders through a neurological examination. According to the quality criteria of the studies, 83% of articles were intermediate to high quality. Three studies presented high quality, three studies presented intermediate-high quality, four studies showed intermediate quality, and two studies showed intermediate-low quality.

When reviewing the outcome related to exposure to pyrethroids, it can be observed that four studies did not find effects or associations with neurobehavioral, neuromotor, or neurocognitive outcomes. However, one of the studies presents a limitation of the low sample size (Lu et al., 2009), a manuscript reports that it measured the exposure with self-records of pesticide use without a control group (Zhang et al., 2018), and another research only measured historical self-report recalled by the patients themselves, which may lead to memory bias (Weisskopf et al., 2013).

Of the eight studies that found neurological effects (66.6%), four studies reported that exposure to pyrethroids impacts neurological or neurobehavioral functioning in pesticide applicators (Huang et al., 2016; Hansen et al., 2017; Ismail et al., 2018; Shrestha et al., 2018).

One of the studies with pesticide applicators from Saudi Arabia (Ismail et al., 2018) examined neurological symptoms, neurobehavioral performance, sociodemographic variables, and their relationship with Khat consumption and exposure to pyrethroids using a questionnaire and measuring the exposure to organophosphates with cholinesterase levels. They observed poorer performance of an applicator in the executive and motor speed/coordination functions (symbol digit test latency; tapping (TAP) non-preferred hand; and TAP alternating hands). Khat chewing was associated with memory deficits and motor speed/coordination functions.

Another work that evaluated the factors associated with dream enacting behaviors among a group of applicators from the United States (Shrestha et al., 2018) found significant associations between dream enacting behavior (DEB) during REM sleep, with the application of pyrethroids (OR = 1.3; CI = 1.1–1.5), specifically in insecticide permethrin linked to poultry/livestock (OR = 1.4; CI = 1.2–1.6).

In Bolivian pesticide applicators, Hansen et al. (2017) estimated the association between exposure to pyrethroids and neuromotor and neurocognitive performance in workers. A high-level pyrethroid exposure was associated with reduced neurocognitive performance [adjusted β = −1.344 (−2.224 to −0.464)]. In neuromotor performance, no association was observed with the application of pyrethroids.

In Chinese applicators, Huang et al. (2016) evaluated exposure to pyrethroids and conventional nerve conduction, finding only an association with the decrease of the ulnar nerve conduction following the short-term exposure.

In agricultural workers in India with high and medium chronic exposure to pesticides, Kori et al. (2018b) found that symptoms of stress (*p* < 0.01) and anxiety (*p* < 0.05) were observed mainly in those workers who store pesticides (pyrethroids and others) at home. Among these workers, pyrethroids were the most widely used pesticide, with 7% of farmers using a very dangerous pyrethroid and 34% a moderately dangerous one. The adverse health effects were greater in agricultural workers with high chronic exposure than those with medium exposure to pesticides (Depression: 19.7% high vs. 10.3% medium; Anxiety: 50.7% high vs. 32.7% medium; Sleep problem 41.6% high vs. 36.2% medium). It is noteworthy that only bivariate chi-square analyses were applied to establish associations between exposure and symptoms in this study.

Another research conducted with part-time or full-time male and female agricultural workers in the Philippines (Lu, 2009) found that most farmers used pyrethroids (71.1% full-time and 73% part-time), but full-time workers also used organophosphates. Both full-time workers (5.22%) and part-time workers (8.63%) presented an abnormal neuromotor diagnosis. Three out of 139 part-time farmers had cerebellar dysfunction. Therefore, full-time or part-time farmers were equally exposed to pyrethroid pesticides and had similar neuromotor symptoms. A critical weakness of this work was that it did not have a comparison group without exposure to pesticides.

On the other hand, only one investigation with South African women (Motsoeneng and Dalvie, 2015) reported exposure to pyrethroids through specific metabolites concentrations in urine. Neurological symptoms were measured through the Q16 questionnaire. This article reported that the prevalence of all Q16 symptoms was higher among farm women than town women. Three Q16 symptoms (problems with buttoning, reading, and notes) were significantly positively associated with three pyrethroid metabolites (cis-DCCA, trans-DCCA, and DBCA); however, associations may be due to chance as multiple comparisons were made. The strongest association for a pyrethroid metabolite was between problems with buttoning and DBCA (odds ratio = 8.93; 95% CI = 1.71–46.5).

Finally, a study with children of agricultural workers living near or working in large and small-scale banana plantations in Costa Rica (van Wendel de Joode et al., 2016) compared the results in neurobehavioral and neurotonic functioning with the results in urine metabolites of pyrethroids from three communities. Higher 3-PBA concentrations were associated with poorer processing speed scores, especially in girls (β = −8.8; 95% CI: −16.1, −1.4).

## Discussion

Although the studies addressing this issue are scarce, showing the lack of research that evaluates epidemiologically pyrethroid pesticides exposure and neuro outcomes, they confirm the health risk involved for adults at work and children indirectly exposed by the agricultural occupation of their parents.

The results of this systematic review show that in 66% of the studies, agricultural workers or their children exposed to pyrethroid pesticides have a higher risk of presenting difficulties in their neurobehavioral, neuromotor, or neurocognitive performance, mainly associated with attention and processing speed problems. Other neurological alterations associated with sleep and the response of the peripheral nervous system are also observed, which affects neurobehavioral performance in areas of motor coordination. Simultaneous exposure to other dangerous pesticides, such as organophosphates and herbicides, increases the risk of suffering from neurobehavioral disorders and other health problems.

Other studies have investigated the exposure to pyrethroids and neurobehavioral effects in the general population, specifically children (Wang et al., 2016; Viel et al., 2017), and compared the levels of specific metabolites of pyrethroids in schoolchildren with other similar research, showing that the risk is also increasing in the non-occupational population (Muñoz-Quezada et al., 2020). The studies included in this review highlight the relevance of strengthening research of this type of pesticide, which has been offered from the agrochemical market as the supposedly less harmful alternative for insect pests control and elimination in rural or urban areas.

Furthermore, in recent years, the sale of pyrethroids has been increasing, and it is presented as the solution to replace pesticides that are more hazardous to human health (e.g., organophosphates). For more than 20 years, different studies have shown how chronic exposure to low doses of organophosphates over time causes neurobehavioral and neurodevelopmental health issues. Also, there is evidence that they are carcinogenic, genotoxic, and are related to immunological and reproductive alterations, among others, affecting the quality of life of children and adults and polluting the environment (Alavanja and Bonner, 2012; Muñoz-Quezada et al., 2016; Zúñiga-Venegas et al., 2020).

The quality of most of the research conducted in this field (83% intermediate or high quality) and the evidence collected provide information that confirms the risk for neurobehavioral health in agricultural workers due to occupational exposure to pyrethroids. More research is required addressing this subject that evaluates pyrethroids exposure over time through biomarkers and the neuro outcomes with validated neurobehavioral and neuromotor tests, besides evaluating the effect of simultaneous exposure to other hazardous pesticides, such as organophosphates. The relationship with other neurotoxins could be addressed through the real-life risk simulation (RLRS) approach as a complementary instrument aiming to reduce clinical studies with animals and the high cost of longitudinal studies with human populations to accelerate evidence delivery that allows for prompt actions and policies from the authorities to regulate the use of pesticides (Margina et al., 2019; Tsatsakis et al., 2019). Assuming that the use of pyrethroids is increasing considerably and faster than the scientific evidence concerning the effects of its use for human health and the environment, it is suggested as a precautionary principle to regulate more strictly the sale of pyrethroids and other pesticides, train workers on its safe use, and monitor for possible risks to human health.

In addition, it would be advisable to conduct longitudinal studies that allow for monitoring the neurodevelopment in children population in occupationally exposed rural communities from an early age. That would allow eventually to develop timely interventions and guide decision-makers in the restrictions, monitoring, and respective regulations needed to protect exposed, vulnerable communities that already show difficulties in their development, learning, and well-being.

On the other hand, it is a priority to train agricultural workers, communicating the evidence about the health hazards posed by not using personal protection elements (PPE) or the possible effects that the risk behaviors of applicator can cause during the application and mixing of pesticides. It should be encouraged that the participation of communities to collaborate with the interventions designing and planning, so they fit well with their own needs and culture. Furthermore, it should be promoted to involve them actively in the discussion of regulatory measures for better agricultural practices that allow for clean production in the agri-food industry.

Regarding the assessment of neuro outcomes, it is advisable to incorporate, in future epidemiological investigations brain functioning measures as EEG recordings or functional magnetic resonance imaging (fMRI). These measures have the advantage of allowing for studying the brain activity to determine with greater precision, together with the classic neurobehavioral and neurocognitive tests, the impact of chronic exposure to pyrethroids in humans. Some of these neuroimaging measures have already been used in recent studies that evaluated the effects of organophosphates exposure on brain structure (Rauh and Margolis, 2016) and cognitive functioning of children and adolescents (Rauh et al., 2012; van den Dries et al., 2020). Finally, we assume that it would also be relevant to generate research that investigates the mechanisms linked to genotoxicity and the development of cancer and neurodegenerative diseases, such as the study of MicroRNA alteration due to occupational exposure to pesticides and Parkinson's disease (Aloizou et al., 2020; Costa et al., 2020).

## Data Availability Statement

The original contributions presented in the study are included in the article/supplementary material, further inquiries can be directed to the corresponding authors.

## Author Contributions

BL prepared and carried out the bibliographic searches. MM-Q and BL screened articles for inclusion and extracted data. MM-Q and BL critically reviewed the important intellectual content and approved the manuscript. All authors contributed to the article and approved the submitted version and developed the idea for the review and wrote the manuscript.

## Conflict of Interest

The authors declare that the research was conducted in the absence of any commercial or financial relationships that could be construed as a potential conflict of interest.
